# Supporting the decision to perform molecular profiling for cancer patients based on routinely collected data through the use of machine learning

**DOI:** 10.1007/s10238-024-01336-w

**Published:** 2024-04-10

**Authors:** Julia Kasprzak, C. Benedikt Westphalen, Simon Frey, Yvonne Schmitt, Volker Heinemann, Theres Fey, Daniel Nasseh

**Affiliations:** 1 Comprehensive Cancer Center (CCC Munich LMU), LMU University Hospital Munich, Pettenkoferstraße 8a, Munich, Germany; 2 Roche Pharma AG, Grenzach-Wyhlen, Germany; 3 German Cancer Research Center (DKFZ), German Cancer Consortium (DKTK, Partner Site Munich), Heidelberg, Germany

**Keywords:** Decision support, Machine learning, Imbalanced data, Next-generation sequencing, Molecular tumor board, Health informatics

## Abstract

**Background:**

Personalized medicine offers targeted therapy options for cancer treatment. However, the decision whether to include a patient into next-generation sequencing (NGS) testing is not standardized. This may result in some patients receiving unnecessary testing while others who could benefit from it are not tested. Typically, patients who have exhausted conventional treatment options are of interest for consideration in molecularly targeted therapy. To assist clinicians in decision-making, we developed a decision support tool using routine data from a precision oncology program.

**Methods:**

We trained a machine learning model on clinical data to determine whether molecular profiling should be performed for a patient. To validate the model, the model’s predictions were compared with decisions made by a molecular tumor board (MTB) using multiple patient case vignettes with their characteristics.

**Results:**

The prediction model included 440 patients with molecular profiling and 13,587 patients without testing. High area under the curve (AUC) scores indicated the importance of engineered features in deciding on molecular profiling. Patient age, physical condition, tumor type, metastases, and previous therapies were the most important features. During the validation MTB experts made the same decision of recommending a patient for molecular profiling only in 10 out of 15 of their previous cases but there was agreement between the experts and the model in 9 out of 15 cases.

**Conclusion:**

Based on a historical cohort, our predictive model has the potential to assist clinicians in deciding whether to perform molecular profiling.

## Introduction

The concept of precision oncology is based on the fact that cancers can be characterized by specific biomarkers.

Based on comprehensive molecular profiling of an individual’s cancer, precision oncology aims to assign specific molecularly guided treatment options to patients. Recent advances in NGS and the advent of multiple innovative-targeted agents have put the concept of precision oncology further into the spotlight. However, since resources are limited, sustainable ways to implement precision oncology are needed to increase the number of patients potentially benefitting from the combination of molecular profiling and targeted treatment [1].

In the clinical setting, results of molecular profiling are often discussed in so-called MTBs [2, 3]. While the targeted approaches are still experimental in some cases, their effectiveness has already been shown for some types of cancer (e.g., lung cancer) [4, 5]. However, while the potential of precision oncology is becoming more evident, there is limited guidance to determine which patients should receive molecular profiling and be discussed in an MTB [6, 7]. Currently, most of the patients discussed in MTBs have exhausted all standard therapeutic options. This approach might not be the most suitable in all instances, as some patients benefit from up-front testing, while others do not benefit from testing at all. Irrespective of this consideration, inexperienced physicians may encounter challenges in identifying the appropriate subset of patients, particularly those who have exhausted conventional treatment options, for potential inclusion in molecularly targeted therapy initiatives.

Here, we present a prognostic model for predicting the likelihood of the necessity to perform molecular profiling based on routinely collected data and the historical decisions of experts in molecularly targeted therapy regarding patient inclusion into molecular profiling. Our model might function as a decision support system and could support existing inclusion criteria for MTB patients. As the local tumor documentation and molecular database of the Comprehensive Cancer Center at Ludwig Maximilian University of Munich (CCC Munich^LMU^) provided the basis for the development of this model, this also highlights the secondary use capabilities of routinely collected clinical data while also discussing potential and limitations of such data sources.

## Materials and methods

Disclaimer: according to the Bavarian hospitals act [8], all analyses were conducted on site in LMU hospital’s own IT infrastructure. At no point, aside from aggregated/anonymized results, was data transferred out of the hospital. All analyses were conducted using Python 3.8.8. For clarity: within the section of Material and methods as well as Results we used the term NGS test in the model synonymously to performed molecular profiling.

### Data cleaning

The first step was to identify and prepare the relevant data. Two datasets, LMU’s local tumor documentation dataset (CREDOS—cancer retrieval evaluation and documentation system) [9, 10], as well as a custom MTB database, served as source data.

While the local tumor documentation at CCC Munich^LMU^ contains more than 46.000 (19.07.2022) tumor entries, only a fraction of those have been discussed by the MTB (*N* = 1834, 19.07.2022). The MTB cases are labeled with a specific flag in the CREDOS database and have additional information—e.g., the occurrence of pathogenic alterations.

As the tumor documentation is complex (more than 2000 data fields), the quality of its contents can be challenging, as is the case with most routine data in general. To improve this situation, based on discussions with data experts of the CCC, the data contents were restricted and filtered for further analysis. Only those cases primarily treated at the CCC are referred to as primary cases (according to OnkoZert guidelines [11]). About 63% of the CREDOS cohort consists of primary cases. Non-primary cases were not considered for the next steps, as they typically lack data completeness. Another way to improve the data quality was to only include cases with a diagnosis date after 01.01.2016. According to the Center, this decision was based on the introduction of new data standards, which were imposed due to new regional laws (Bayerisches Krebsregistergesetz (state law on the Bavarian cancer registry), [12]). This improved the completeness for many data categories.

Furthermore, a filter was set to exclude benign tumors, defined as those beginning with a ‘D’ code in the ICD-10 classification—e.g., D17—benign lipomatous neoplasm [13]. In addition, those patients with two or more tumors were removed because the MTB database contained only patient IDs but no tumor IDs; hence, the link to CREDOS (which also contains individual tumor IDs) would have been ambivalent. Finally, we considered those patients who received only one NGS test.

While CREDOS contains most of the clinical information about a tumor case, some of the data is difficult to process. For example, chemotherapy substances have been documented quite heterogeneously. For this reason, we translated substance names into standardized ATC codes—e.g., Cisplatin → L01XA01 [14]—which facilitated further steps.

Despite the presence of information regarding patient mutations in the MTB database, the primary aim of this study is not to predict mutations. Instead, it focuses on facilitating the decision-making process for including patients in an MTB. Hence, only this information from the MTB database was necessary for the study’s objectives.

### Selection and description of the features

After restricting the number of patients, we then selected the features of interest. These features were selected according to interviews with local data experts as well as MTB experts. Some features were added from other data sources, in particular information about the NGS test performed, which came from the molecular database, and additional information on transport data, which came from the hospital admission or cancer incidence calculated from The German Centre for Cancer Registry Data [15]. Table 1 shows the final selection of features.

**Table 1:** Final selection of features with possible values

**Demographics**	**Values**
Gender	Female, male
Age at diagnosis	1–100
**Anamnesis**	**Values**
Cancer entity	Breast, lung, pancreas, prostate, colon, biliary tract, others
UICC staging	I, II, III, IV
TNM staging	Tis, Ta, T0-T4, N0-N3, M0-M1
Grading	G1, G2, G3, G4, unknown
Metastases present	Yes/no
Metastases	Number of detected metastases
ECOG performance status	Unknown, 0, 1, 2, 3, 4
**Progress**	**Values**
UICC staging	I, II, III, IV, unknown
Grading	G1, G2, G3, G4, unknown
TNM staging	Tis, Ta, T0-T4, N0-N3, M0-M1
Metastases present	Yes/no/unknown
Metastases	Number of detected metastases
ECOG performance status	unknown, 0, 1, 2, 3, 4
Revaluation	Remission, progression, recurrence, stable, mixed response, unknown
Situation	Curative, palliative, unknown
Survival status	Death due to tumor, death independent of tumor, cause of death unknown, lost to follow-up, live
**Treatments**	**Values**
Type of therapy: • Surgery, • Radiotherapy, • Medical therapy, • Medical therapy with personalized treatment, • Others	Number of respective therapy
Therapy phase	Primary, secondary
Therapeutic goal	Curative, palliative, neoadjuvant, adjuvant, diagnostic, unknown
**Others**	**Values**
Quarters since the first event date	Number of quarters since the first event date in the patient record
Transport information: • By foot, • Bed, • Stretcher, • Material transport, • Wheelchair, • Heavy duty bed, • Heavy duty wheelchair	Number of respective transports
NGS test performed	0, 1
Frequency of cancer (relative cancer incidence) at given age, gender, and entity relative to gender and entity cohort	Relative frequency between [0, 1]

The selected features can be divided into 5 categories; see Table 1. The first category contains the demographic data *gender* and *age at diagnosis*, which are examples of the features that were only added after discussion with the MTB experts, as they pointed out that they are very important in deciding whether a patient should receive an NGS test. The younger the patient, the higher the probability that a gene mutation is the cause of the tumor [16–19].

The next category is anamnesis, which includes information about initial findings, such as *initial diagnosis* according to the ICD-10 classification and *number of initial metastases*, as well as *UICC* [20], *TNM* [21], and *grading*, which are common staging classifications used to describe the severity of a case. *ECOG performance status* describes the physician’s impression about a patient’s well-being as a value between ‘0’ and ‘4’ [22]. ECOG was one of the fields identified to have insufficient data quality/completeness.

The following category includes those features that describe the patient’s progress. Here, some features from the previous category were reused for the model. *Staging*, *ECOG* and *number of metastases* can change during the course of the patient’s disease, and such information might be important for further treatment. *The revaluation* feature identified the status of a tumor at different timestamps in terms of remission, progression, recurrence, stable and mixed response. The next feature showed whether the patient was in a curative or palliative *situation*, while the *survival status* provided information about the date of death or the last vital date. Acquiring vital data is often a problem, but the primary cases in CREDOS usually have a follow-up rate of over 80%. This high follow-up rate is due to the CCC’s own efforts as well as supporting information from the Bavarian Cancer Registry [23].

The next category includes treatment features such as *the type of therapy*, which is differentiated into surgery, radiation therapy, medical therapy with and without personalized treatment, and ‘others’. The next feature, *the therapy phase*, described whether the therapy performed was in the primary or secondary phase. The last feature from this category was used to describe *the therapeutic goal*, and it had the following values: curative, palliative, neoadjuvant, adjuvant or diagnostic.

The last category includes features that do not fit into any of the above categories. The first feature is information on *how many quarters* have passed since the patient’s first event (e.g., initial diagnosis) until the next event (e.g., until the performing of an NGS test or the start of personalized therapy). *The transport data*, which give information similar to that of the ECOG in that they indicate a patient’s condition, were added after consultation with the MTB experts in order to solve the problem of the ECOG’s lack of completeness. When cancer patients are transported at the hospital, they are either coming by foot or are moved (e.g., in a wheelchair or bed). While these data are not as precise as the ECOG, it has been documented for many more timestamps. If *an NGS test had been performed* is another feature of this category. The value ‘0’ was given when the test was not performed in a given quarter, and the value ‘1’ was give when it was performed in given quarter. The last feature is *the cancer incidence,* which was calculated using age at diagnosis, gender and tumor entity in relation to the epidemiological cohorts represented in The Centre for Cancer Registry Data, resulting in individual incidence-values ranging from 0 to 1 for each case.

### Additional feature preparation

After selecting the features, some of them had to be prepared according to the requirements of the prediction model. In some cases, we trimmed down complexity and reduced dimensionality by aggregating some of the data into generalized groups.

The generalization into groups was applied to the initial diagnosis (ICD-10 codes) of the tumor documentation, which was grouped into the following subgroups: breast, lung, pancreas, prostate, colon, biliary tract and others. Generalization was also used for UICC, TNM, grading, and revaluation, reducing the dimensionality of their categories by about half.

Another method of preparing some of the features was to represent their value set via the count of their occurrences in a quarter instead of their actual value. For example, instead of storing two individual surgery dates, a count of ‘2’ was documented. To illustrate this better, the aforementioned procedure is shown in the ‘Type of therapy—surgery’ column in Table 2. This was done analogously for the transport data as well, which showed how often the patient moved (e.g., by foot or in a wheelchair) during a given quarter.

**Table 2:** Extract of the input format for the prognostic model. This example shows how some features, like the type of therapy, were aggregated by counting occurrences during a quarter instead of listing each date value in order to decrease complexity.

Patient ID	Quarter	NGS test	Cancer entity	Age	Gender	…	UICC	Metastases	Revaluation		Type of therapy—surgery	…
12345678	2018-Q3	0	Others	76	F	…	IV	No	Stable	2		…
12345678	2018-Q4	0	Others	76	F	…	IV	No	Stable	0		…
12345678	2019-Q1	0	Others	76	F	…	IV	No	Stable	1		…
12345678	2019-Q2	0	Others	76	F	…	IV	No	Stable	0		…
12345678	2019-Q3	0	Others	76	F	…	IV	No	Stable	1		…
12345678	2019-Q4	1	Others	76	F	…	IV	Yes	Progression	0		…
…	…	…	…	…	…	…	…	…	…	…		…

### Creation of quarterly panel

The cohort with the selected features was reorganized in a quarterly panel. Each patient in each quarter between Q1-2016 and Q3-2021 was represented as a row. Each row contained information on the selected features. Table 2 gives an impression of the given data model.

Since multiple events per feature can occur in one quarter, we had to aggregate this information to be represented as a single row. For some features (therapies, transport data, and number of detected metastases), this was explained above. For all other features, only the last available value of a feature in the given quarter was taken into consideration and imputed into the quarterly panel. For example, if a patient’s ECOG was documented with a value of ‘0’ at first but then with a value of ‘1’ at a later point in the same quarter, the value of ‘1’, as the latest documented value, was used for further analysis.

The outcome is named ‘NGS Test’ (see Table 2) and indicates whether a NGS test was performed in the given quarter (‘NGS Test’ = 1) or not (‘NGS Test’ = 0) for the respective patient. The prediction model described in the following section is used to estimate this outcome on the first day of each respective quarter, given the most recent available information about the patient—i.e., aggregated information from the previous quarter. For example, a patient’s grading from 15 January (Q1) is used as a feature for predicting the necessity of an NGS test in Q2.

The next step to obtain the best possible data model was to replace missing values with three options. First, if no value for grading was documented in a given quarter, the last available grading value from the previous quarters was rewritten. Second, in the case of no therapy per quarter, the value ‘0’ was used as a replacement for the missing value. Third, for other features, the missing values were labeled as ‘unknown.’

### Implementation of the model

The created data model with all selected features was split into training and test data sets. The different splits for the training and test sets were not described. Only the best split was shown in the work, which resulted in the best model performance. The test set contained data from the last available quarter (Q3-2021), while the training set contained all other quarters. It is important to note that our dataset is imbalanced, and the implications of this are discussed in detail later on. This imbalance requires careful consideration and planning in the analysis steps that will follow.

To estimate the probability that an NGS test would be performed for patients during the test quarter, a machine-learning algorithm was trained. It can identify the most important of the various prepared features and automatically approximate the clinically complex function between these features and the target variable.

For this paper, LightGBM, a gradient boosting framework that uses tree-based learning algorithms [24], was used. Gradient boosted trees are a potent machine learning algorithm that typically yields superior performance compared to alternative methods, such as neural networks or simple statistical models like logistic regression, when applied to tabular data [25]. Furthermore, this algorithm is highly recommended for imbalanced classification tasks [26].

The LGBMClassifier is a class of the framework that can predict the probability of class memberships—e.g., the probability for ‘NGS test’ vs. ‘no NGS test’—and offers many hyper-parameters that can be tuned to improve prediction accuracy.

Cross-validation was performed, using GridSearchCV [27] to find the optimal combination of hyper-parameters for the model. In particular, we decided to tune the following hyperparameters: the learning rate, the number of boosted trees, the number of leaves in each tree and the minimum number of observations per leaf in the training data [28].

### Evaluation of model performance

The final step in building the prediction model was an evaluation to check the performance of the obtained model.

Since the model predicts a continuous probability, its outputs have to be mapped to one of two decisions or classes by using a probability threshold. The model in this paper predicted the probability that an NGS test would be performed in the given quarter; thus, it returned a value in the interval [0, 1] for each observation. In order to classify the observations, a probability threshold for the model at hand was set: values below this threshold were interpreted as a recommendation for ‘no NGS test’, while values above were interpreted as a recommendation for ‘NGS test.’ In general, specifying an optimal threshold does not change the method of probability estimation, but it does affect the method of case classification. The selection of an appropriate threshold value is critical for achieving the desired objectives in classification tasks. The default threshold value is conventionally set to 0.5; nevertheless, this value may not always be appropriate, especially for models based on imbalanced data [29]. In such cases, the model may not achieve high accuracy, or it may generate a large number of false positives, resulting in elevated costs within the confusion matrix. Therefore, it is imperative to adjust the threshold value as necessary to optimize model performance.

**Fig. 1:**
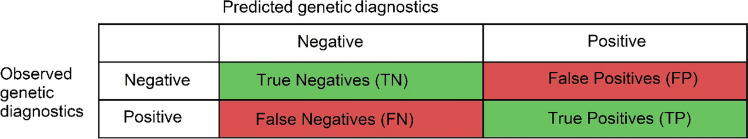
General setup of a confusion matrix

Positive and negative classifications can be represented using a confusion matrix (see Fig. 1).

A negative classification means that the event does not occur (value ‘*N*’), while a positive classification indicates the occurrence of the event (value ‘*P*’). Figure 1 summarizes the decisions made by the model in relation to the actual values. The correct decisions are marked in green: true negatives (TN) and true positives (TP). The red identifies errors: negative cases classified as positive by the prediction model (false positives—FP) and positive cases classified as negative (false negatives—FN) [29, 30]. FP for our model indicated that patients who did not receive the NGS test were assigned a test, while FN indicated that the test was underestimated—i.e., patients who actually had the test were classified as patients without the test. We aimed for the values for these errors to be as low as possible.

Appropriate evaluation metrics are crucial for accurately assessing model performance based on the confusion matrix. These metrics are used to compare different models and estimate the impact of manipulating the classification threshold. While there are numerous evaluation metrics available, in this study, we focused solely on those that best reflect the true nature of imbalanced data.

In this study, the model was evaluated using the receiver operating characteristics (ROC) curve with its associated AUC for both the training and test sets. [29, 30].

As an alternative to the ROC curve, we calculated the Precision-Recall curve, which can provide better results for imbalanced data.

Sensitivity, represented by formula (1), measures how well the positive class (‘NGS test’) was predicted. A higher sensitivity value indicates better positive class prediction. This metric might be particularly important in medical data, where it is desirable to minimize the number of missed positive cases [31, 32].

1 $${\text{Sensitivity}}/{\text{recall}}/{\text{TPR}} = \frac{{{\text{TP}}}}{{\left( {{\text{TP}} + {\text{FN}}} \right)}}$$

Another important metric is specificity, representing the percentage of correctly classified negative cases (‘No NGS test’), represented by the formula (2) [29]. In big health datasets, it is important to detect rare but significant cases to measure sensitivity. However, a trade-off between sensitivity and specificity should be considered, as indiscriminately increasing the sensitivity score may result in a higher number of false positives, and thus a low specificity score [31].

2 $${\text{Specificity}} = \frac{{{\text{TN}}}}{{\left( {{\text{FP}} + {\text{TN}}} \right)}}$$

## Results

Following the preparatory steps, the data model was constructed with 14,027 patients, consisting of 440 patients with NGS tests and 13,587 patients without NGS tests. The population differences, with 30 times more patients without NGS test, favor the usage of an imbalanced classification. The data model consisted of 146,034 rows covering all quarters across all patients and 37 columns, which was equal to the number of selected features. The data was split into a training set consisting of 133,598 rows and a test set consisting of 12,437 rows. The number of patients with NGS tests performed was 32 in the test set and 408 in the training set.

The values for the hyper-parameters we chose for modeling were as follows: learning rate = 0.05, number of boosted trees = 75, number of leaves in each tree = 30 and minimum number of observations per leaf in the training data = 100.

**Fig. 2:**
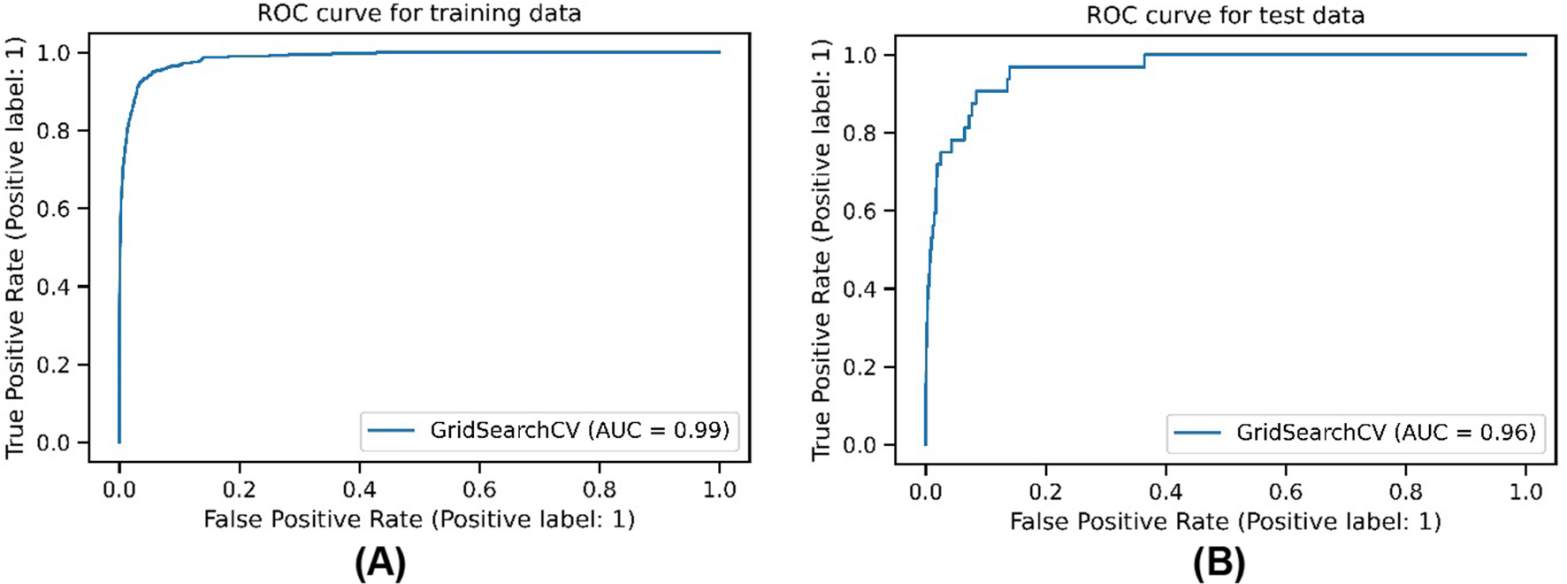
ROC curves with AUC values for: **A** training data and **B** test data

The high AUC scores on the training (AUC = 0.99) and test data sets (AUC = 0.96) indicate that the engineered features are meaningful for deciding whether an NGS test should be performed in the test quarter. The ROC curves with their AUC values for training data and test data are shown in Fig. 2.

**Fig. 3:**
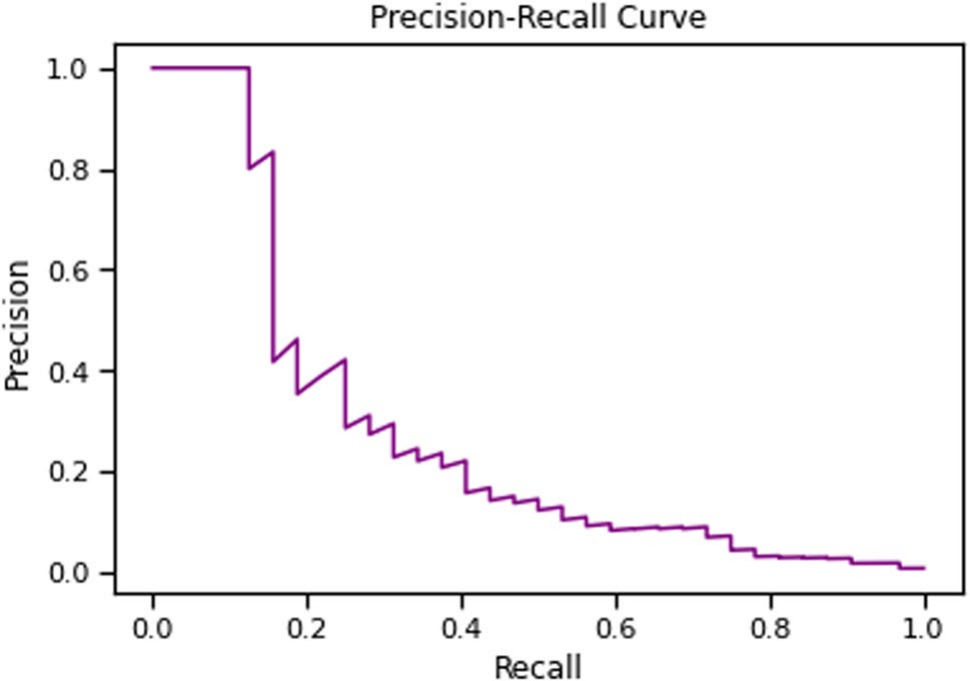
The Precision-Recall curve summarizes the relationship between precision and recall for different thresholds. The model achieved a relatively high precision (precision = 1.0), meaning that it correctly predicted the positive cases in most cases. The trade-off is that the recall was low (recall = 0.09375), indicating that the model captured only a small fraction of all true positive cases in the test set. However, this could be due to the small number of positive cases in the test set. With only 32 patients with molecular profiling in the test set, the model had fewer positive examples to learn from, making it more challenging to accurately capture all positive cases

Additionally, Fig. 3 shows the results of the Precision-Recall curve, with an AUC value of 0.2711.

**Fig. 4:**
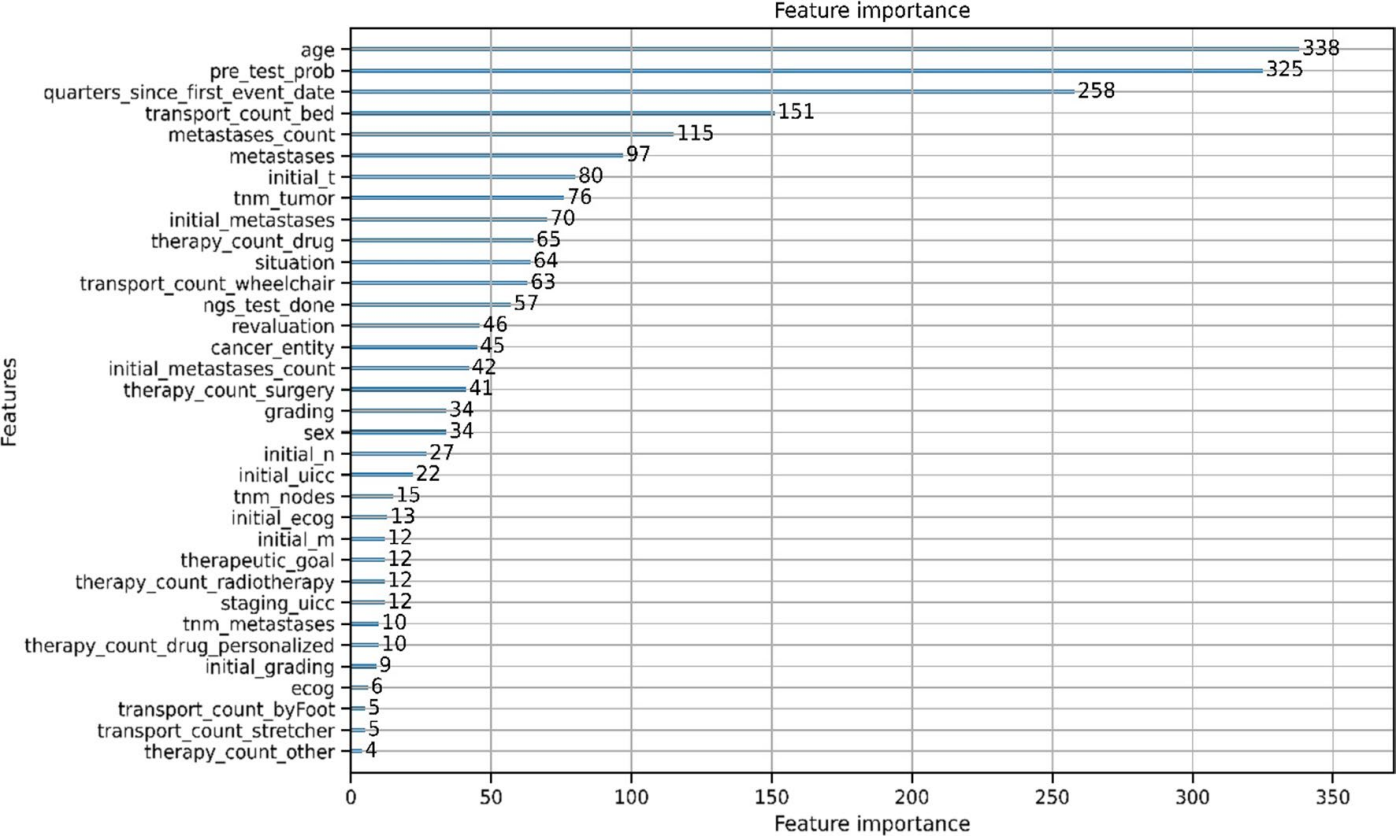
Feature importance

Figure 4 shows the feature importance—i.e., the calculated number of times the feature was used in a model. The higher the value of the feature, the higher the impact on the prediction model. The five most important features were age, cancer incidence, number of quarters elapsed since the first patient event, number of bed transfers and number of metastases. These features accounted for 55% of the use of all features.

**Fig. 5:**
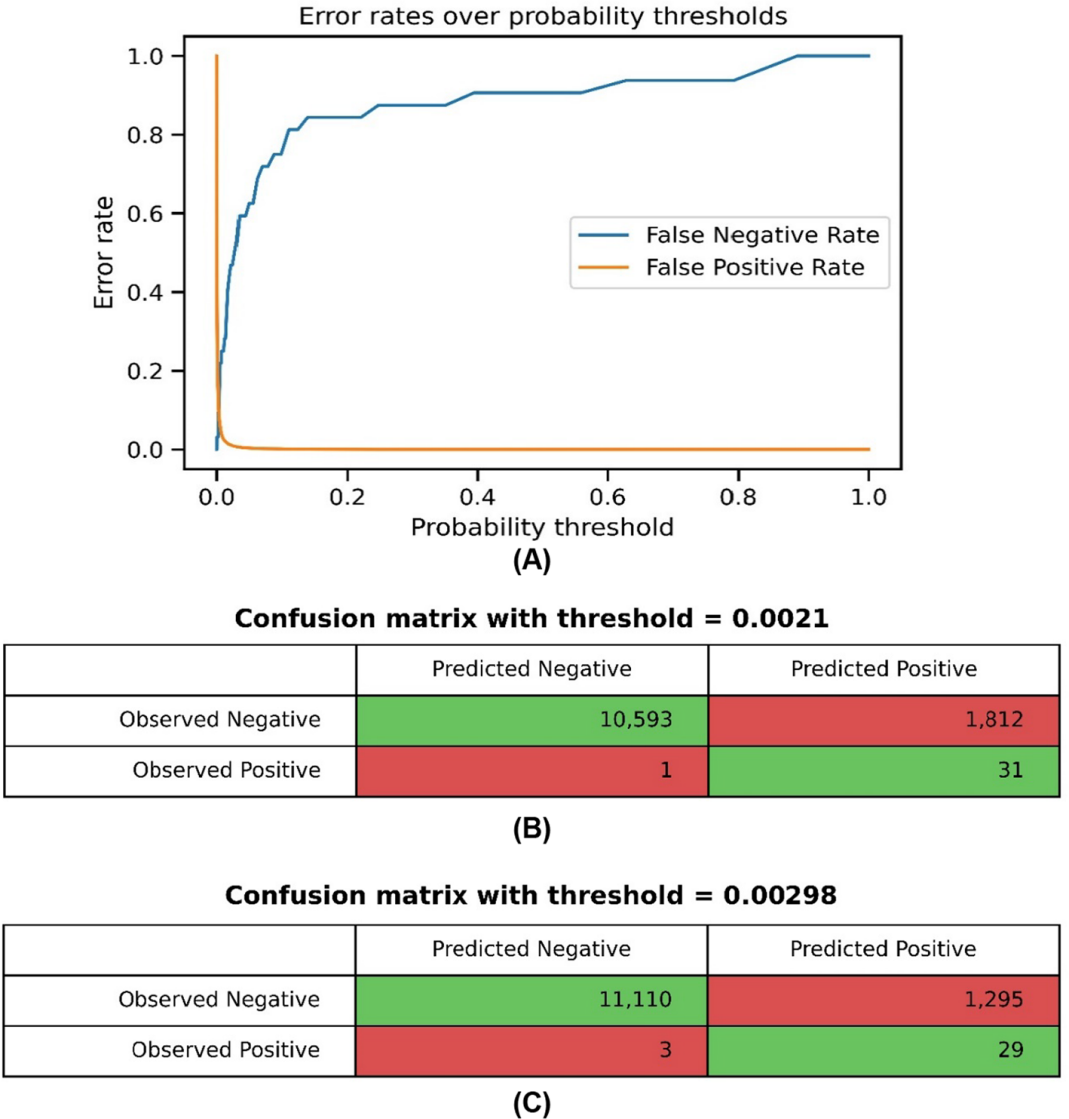
**A** Error rates over probability thresholds. **B** Confusion matrix with the threshold = 0.0021 for maximum of acceptable value of FN 0.5. **C** Confusion matrix with the threshold = 0.00298

The lowest thresholds were 0.0021 and 0.00298 and resulted in equal values of the error rates (FPR and FNR), the first of which was obtained by setting the maximum acceptable value of FN to 0.5. The sensitivity values for both thresholds were: 0.96875 and 0.90625 and the specificity values: 0.8539 and 0.8956. The graph in Fig. 5A shows the values of FPR and FNR for different probability thresholds. The confusion matrices for the calculated thresholds are shown in Fig. 5B and Fig. 5C.

**Fig. 6:**
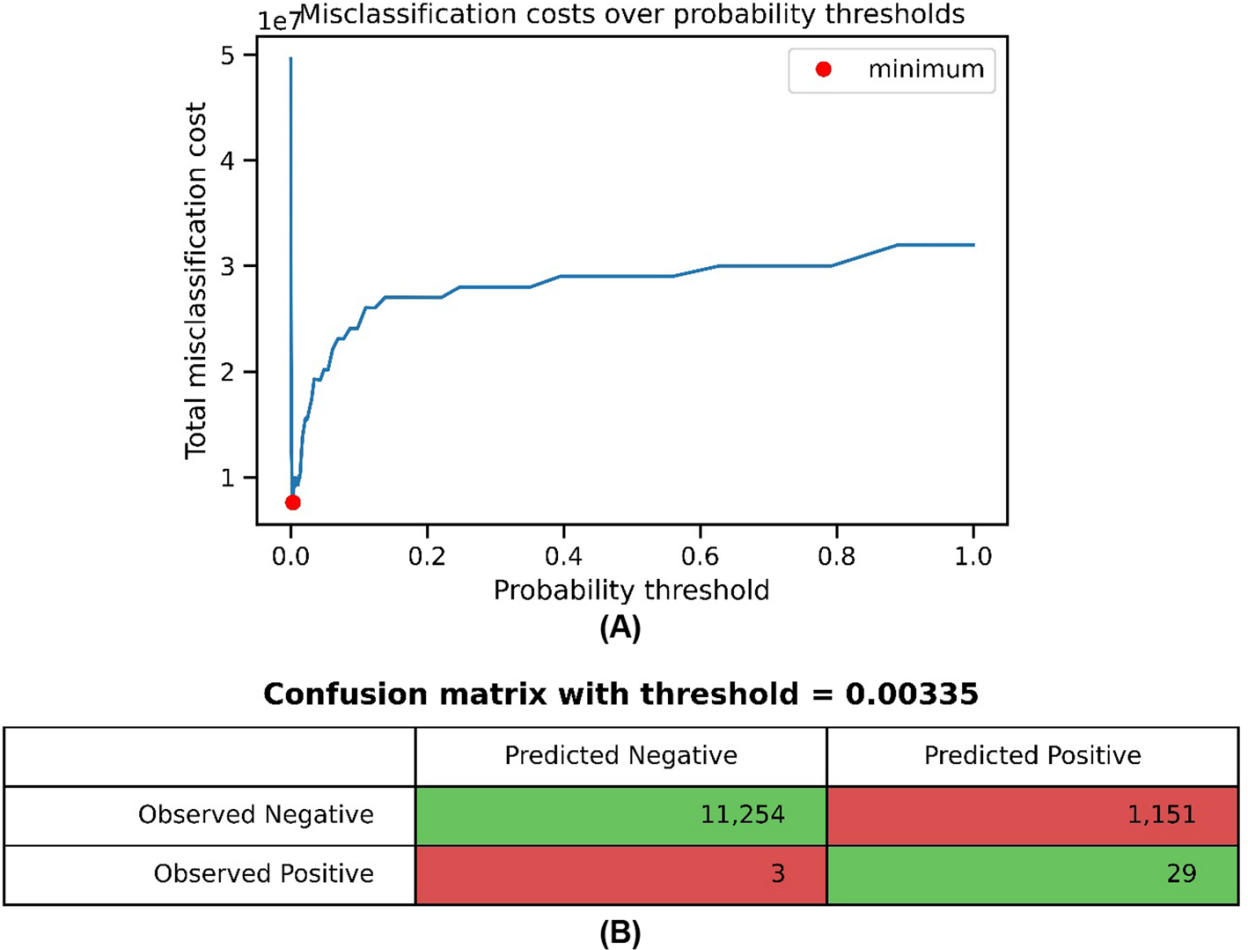
**A** Misclassification costs over probability thresholds. **B** The confusion matrix with the threshold = 0.00335

The other calculated threshold that minimizes the misclassification cost was 0.00335, the sensitivity was 0.90625 and the specificity was 0.9072. The cost of correct classifications (TP and TN) is set as a value of ‘0’, while the cost of misclassifications was set to 1,000,000 for FN and 4,000 for FP. The cost of misclassification was calculated by multiplying each entry in the confusion matrix by the specified cost value [33]. The cost values for probability thresholds and the confusion matrix are shown in Fig. 6.

**Fig. 7:**
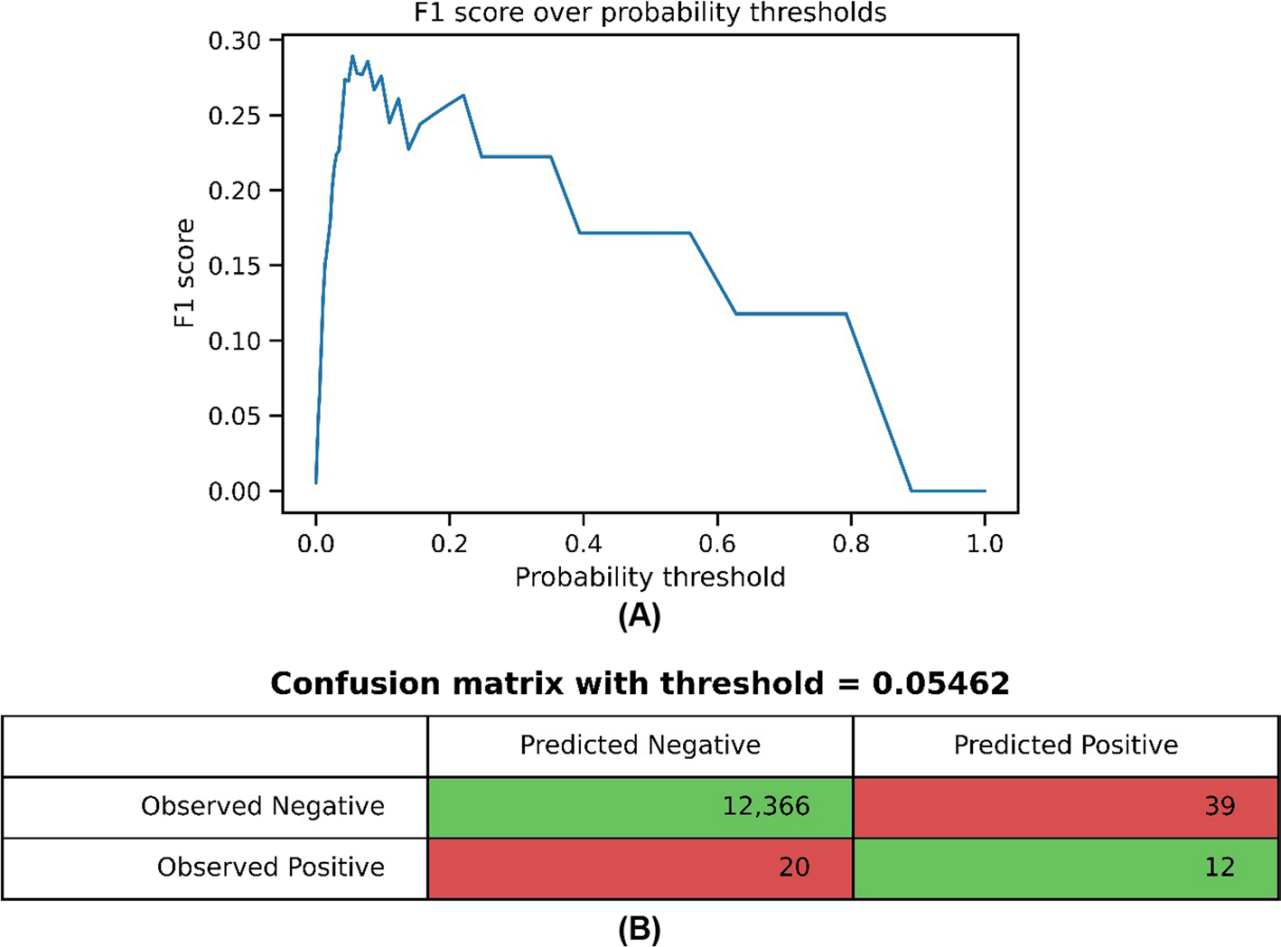
**A** F1 score over probability thresholds. **B** The confusion matrix for the threshold = 0.05462

Figure 7 shows the values of the F1 score for the probability thresholds and the confusion matrix for the threshold of 0.05462 that maximized the F1 score. The sensitivity and the specificity for the calculated threshold were 0.375 and 0.9969.

**Fig. 8:**
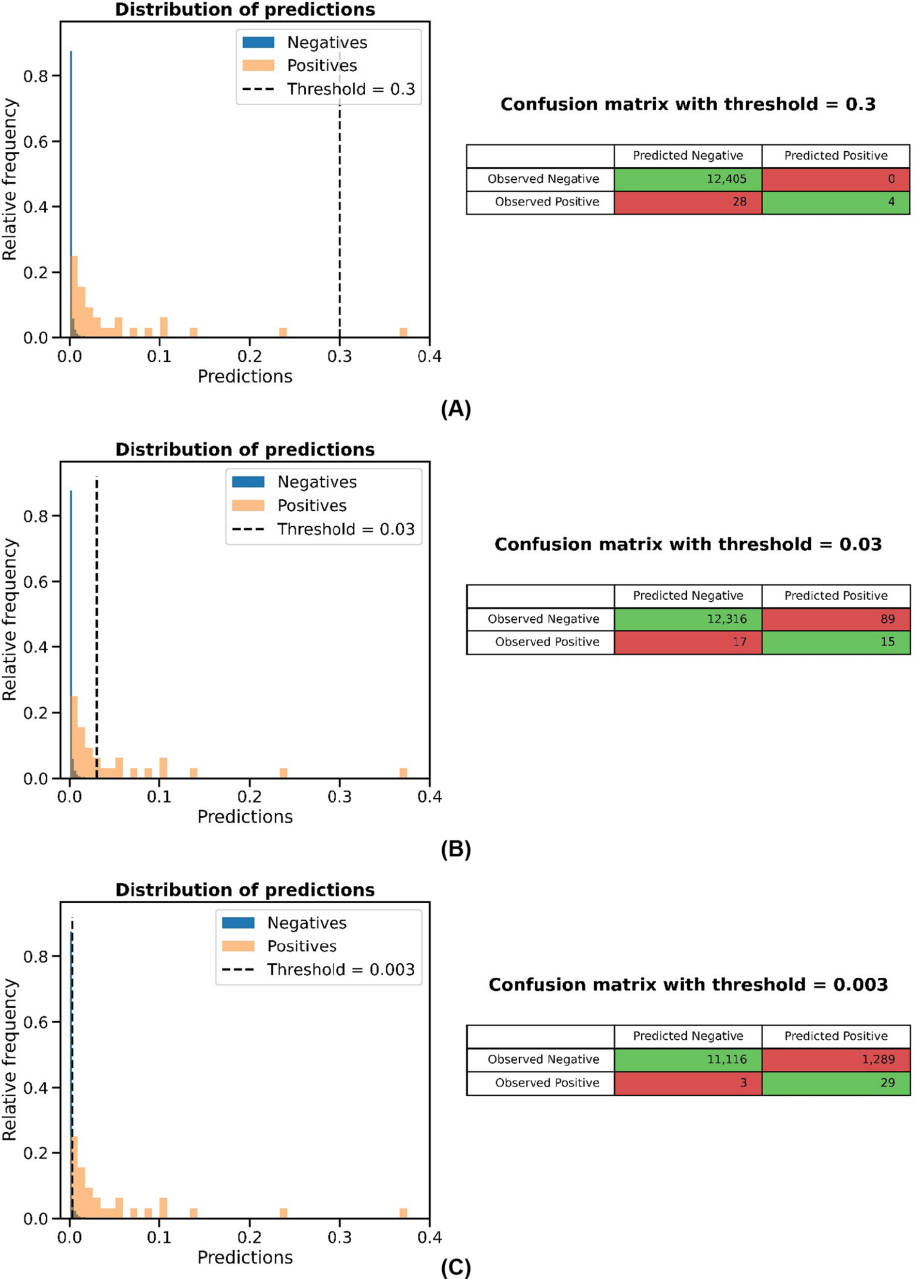
Distribution of predictions and confusion matrices for the following thresholds: **A** 0.3, **B** 0.03 and **C** 0.003

After receiving different classification thresholds, we compared both the distribution of predictions and the confusion matrix for three thresholds as well as the resulting sensitivity and specificity values: 0.3 (0.125 sensitivity/1.0 specificity), 0.03 (0.46875 sensitivity/0.9928 specificity) and 0.003 (0.90625 sensitivity/0.896 specificity). The graphs and matrices are shown in Fig. 8. After discussing the costs of false negatives and false positives with MTB experts, a threshold value of 0.003 was chosen. This selection favors false positive results and, thus, overestimation of NGS tests.

To assess the model’s performance, individual case vignettes of past cases were assessed with MTB experts. For the discussion, we presented 8 pancreatic cancer patients and 7 biliary tract cancer patients, all with engineered features but none with the actual or the model’s test decision. The cases were selected to cover all fields of the confusion matrix equally, leading to a model accuracy of approximately 50%, by design, on this subset of patients. To be specific, the model predicted 8/15 cases correctly. In terms of overall model accuracy, the selected threshold of 0.003 resulted in an accuracy rate of 89.61% (*N* = 11,145/12,437). However, given the imbalanced nature of the dataset, this metric may not be of significant importance. Instead, measures of sensitivity and specificity, as presented previously, may be more crucial in evaluating the performance of the model.

Based on the provided features, the MTB experts correctly decided 10/15 cases and slightly outperformed the model on this small sample. The MTB experts and the model agreed on 9/15 cases.

## Discussion

Allocation of patients to comprehensive molecular profiling has not been standardized on a regional or national level. As of today, the most common recommendation factors are the patient’s age, physical condition, type of cancer, presence of metastases, and therapies performed so far. It is important to note that this study represents a single-center experience.

The prediction model developed in this work could support experienced physicians in their decision to perform an NGS test or not. It may also help identify patients with a fitting profile who have not yet received NGS and would likely have been missed. Likewise, it could also serve as a clinical decision support tool for outreach areas where there are no MTB practitioners or experts, potentially increasing the rate of inclusion of fitting candidates into an MTB, hence increasing the actionability of the NGS test results.

The utilization of routine clinical data to build machine learning models is common practice. However, one of the most prevalent issues with this approach is an imbalanced classification, which is characterized by a skewness in the class distribution. The majority class, typically referred to as the negative outcome, outnumbers the minority class, which is typically defined as the positive outcome [26, 31].

In our study, we observed a significant imbalance in the number of patients with or without molecular profiling. The minority class, which was represented by patients with molecular profiling, was considerably smaller than the majority class, indicating that the number of NGS tests performed is still relatively low compared to the number of cancer patients. This may be attributed to the fact that conventional treatment methods, such as chemotherapy, radiotherapy, and surgery, are commonly used as first-line therapy in most types and stages of cancer, while personalized therapy is usually reserved for second-line or subsequent lines of treatment [34].

Machine learning models typically assume an equal distribution of both classes and aim to achieve a high accuracy score for the entire model. However, in the case of an imbalanced classification, such an approach would prioritize the majority class and result in low sensitivity toward the minority class. Hence, it is crucial to select appropriate evaluation metrics that focus on the minority class, despite the scarcity of observations in this class. Misclassifying the minority class would be much more costly than the majority class, particularly when using patients’ health data. In our case, misclassifying the minority class would result in a patient not receiving NGS test, even though their characteristics indicate that the test should be performed. Therefore, we advocate for the use of evaluation measures that prioritize the minority class, even though it is challenging to identify such metrics [26, 29, 31].

We investigated the impact of selecting different thresholds on accuracy, sensitivity, and specificity in our model. Our results indicated that choosing a high sensitivity threshold (0.003: 0.90625 sensitivity/0.896 specificity) would improve the identification of potential candidates. However, the cost of performing a large number of tests would be high, making this approach potentially economically unsustainable. Conversely, selecting a threshold with high specificity (0.3: 0.125 sensitivity/1.0 specificity) would minimize false positive results, but the sensitivity would be relatively low, potentially leading to too many missed patients.

Given the actionability and the associated current cost of NGS testing, it appears reasonable to prioritize a threshold with a relatively high specificity. As of today, the cost of current comprehensive molecular profiling varies depending on the specific test and provider, but a typical test can cost several thousand US dollars [35, 36]. In that regard a threshold of 0.03 (0.46875 sensitivity/0.9928 specificity) could allow for the identification of about half of the potential candidates while minimizing the number of unnecessary tests. It is worth noting that selecting a threshold with higher sensitivity may be beneficial when cost is not a major concern. Indeed, the trend observed in genome sequencing suggests that the cost of NGS testing, besides emerging new methods, is likely to decrease further in the foreseeable future [37].

After the MTB experts had validated the model via the case vignettes (using the 0.003 threshold/high specificity), they were positively surprised by the agreement of prediction results. This was supported by the even better results in terms of all cases, as well as the high AUC scores, as presented in the results. Questions may arise regarding whether a model relying on routinely collected tumor documentation data could eventually replace human decision-making. However, it should be emphasized that this system is intended solely as a clinical decision support tool, and its clinical utility needs to be demonstrated through broader testing first.

The data model provides valuable insights into the current patient selection process for comprehensive molecular profiling and case discussion in the MTB at LMU. It has the potential for swift updates to reflect any changes in criteria by incorporating more recent cases. Moreover, in theory, with appropriate local adjustments, the model could be potentially adapted for use in other institutions hosting tumor documentation databases and patient transport data. A necessity for the model to perform is data. In this work, we did not collect clean data comparable to a clinical trial; instead, we used the already available and routinely collected data from LMU’s hospital. Hence, as most sites should be able to supply similar data, the methodology could be transferred to other sites in a federated approach. Data quality is the main issue when working with data collected in clinical routine.

Our feature-importance analysis revealed that the top five rated features were age, cancer incidence, number of quarters elapsed since the first patient event, number of bed transfers, and number of metastases. These parameters seem to wield considerable influence, as they identify patients in a relatively poor clinical condition, frequently having undergone standard therapies to exhaustion. This holds particular relevance for the MTB, which assesses innovative approaches grounded in molecular profiles, typically unsuitable for patients who respond favorably to standard therapies. Surprisingly, the number of bed transfers was rated higher compared to the significantly lower rating of the ECOG score. This could be attributed to the incompleteness of the ECOG data in the tumor documentation mentioned in Sect. “Selection and description of the features”, emphasizing the significance of considering additional parameters that may not always be evident.

We believe that this work highlights that sites should invest resources in improving the quality of their routine data for secondary use [38]. Locally, the CCC Munich^LMU^ already invests resources in improving the data quality of its whole tumor data set and has implemented many plausibility checks and other measures [39]. Incompleteness might be the biggest issue [40] observed with data from LMU. Regarding this work, the tumor documentation data alone was not sufficient, but adding additional data, such as the transport data, as well as various data cleaning steps, improved the performance. The model is expected to benefit from improvements in data quality in terms of specificity and sensitivity.

The potential in routinely collected data has also been acknowledged by other groups in the same field. For example, Kim et al. [41] implemented a prognostic model to predict Heme-STAMP (Stanford Actionable Mutation Panel for Hematopoietic and Lymphoid Malignancies) pathological variants based on electronic health records data.

In general, routinely collected data and, in particular, tumor documentation data have been recognized as a very important source for clinical research, as demonstrated by many large scale projects and initiatives utilizing this data source (the national Network Genomic Medicine (nNGM) [5], the German Network for Personalized Medicine (DNPM) [42], the Molecular Tumor Board Alliance (MTBA) [43], the Bavarian Cancer Research Center (BZKF) [44] or the Medical Informatics Initiative Germany (MII) [45]).

Potential extensions to the dataset could include imaging or biomarker data e.g., from blood and urine. However, due to governance issues, these resources were not available to us in this study.

At this point, we regard our system and the current model as a first prototype that offers potential for improvement, refinement and growth. Still, its application in a real-world environment would need further testing and metrics to prove its benefit in comparison to the traditional decision-making process.

## Conclusion

Here, based on routinely collected data, we present a first pilot for a decision support tool capable of predicting if molecular profiling for a cancer patient should be conducted. The system is based on a model created by machine learning and was validated by expert physicians. The results indicate that in prospect of further and possible improvements, such a system might be implemented into clinical care.

## Data Availability

Due to the amount of inspected features, a full anonymization (as required by the GDPA) seems unfeasible. Hence, raw data and model are not fit to be published alongside the manuscript.

## References

[1] Mateo J, Steuten L, Aftimos P, et al. Delivering precision oncology to patients with cancer. Nat Med. 2022;28:658–65. 10.1038/s41591-022-01717-2. 35440717 10.1038/s41591-022-01717-2

[2] Heinrich K, Miller-Phillips L, Ziemann F, et al. Lessons learned: the first consecutive 1000 patients of the CCCMunich^LMU^ molecular tumor board. J Cancer Res Clin Oncol. 2022. 10.1007/s00432-022-04165-0. 35796778 10.1007/s00432-022-04165-0 PMC9261163

[3] Bourien H, Lespagnol A, Campillo-Gimenez B, et al. Implementation of a molecular tumor board at a regional level to improve access to targeted therapy. Int J Clin Oncol. 2020;25:1234–41. 10.1007/s10147-020-01661-6. 32215806 10.1007/s10147-020-01661-6

[4] Rothschild SI. Targeted therapies in non-small cell lung cancer-beyond EGFR and ALK. Cancers (Basel). 2015;7:930–49. 10.3390/cancers7020816. 26018876 10.3390/cancers7020816 PMC4491691

[5] Büttner R, Wolf J, Kron A, et al. Das nationale Netzwerk Genomische Medizin (nNGM). Pathologe. 2019;40:276–80. 10.1007/s00292-019-0605-4. 31101971 10.1007/s00292-019-0605-4

[6] Mosele F, Remon J, Mateo J, et al. Recommendations for the use of next-generation sequencing (NGS) for patients with metastatic cancers: a report from the ESMO precision medicine working group. Ann Oncol. 2020;31:1491–505. 10.1016/j.annonc.2020.07.014. 32853681 10.1016/j.annonc.2020.07.014

[7] Robson ME, Bradbury AR, Arun B, et al. American society of clinical oncology policy statement update: genetic and genomic testing for cancer susceptibility. J Clin Oncol. 2015;33:3660–7. 10.1200/JCO.2015.63.0996. 26324357 10.1200/JCO.2015.63.0996

[8] Bayerisches Krankenhausgesetz (BayKrG): Art. 27, https://www.gesetze-bayern.de/Content/Document/BayKrG ; 2007 [accessed 7 July 2022], [in German].

[9] Voigt W, Steinbock R, Scheffer B. CREDOS 3.1 ein Baukasten zur Tumordokumentation für Epidemiologische-, Klinische,-Tumorspezifische-und Zentrums-register integriert in das KIS SAP/R3 IS-H. Onkologie 2010;33:52. [in German].

[10] Nasseh D, Schneiderbauer S, Lange M, et al. Optimizing the analytical value of oncology-related data based on an in-memory analysis layer: development and assessment of the Munich online comprehensive cancer analysis platform. J Med Internet Res. 2020;22:e16533. 10.2196/16533. 32077858 10.2196/16533 PMC7195671

[11] OnkoZert, https://www.onkozert.de/ [accessed 7 July 2022].

[12] Bayerisches Krebsregistergesetz (BayKRegG), https://www.gesetze-bayern.de/Content/Document/BayKRegG/true; 2017 [accessed 7 July 2022], [in German].

[13] International statistical classification of diseases and related health problems 10^th^ revision (ICD-10), Chapter II Neoplasms (C00–D48), https://icd.who.int/browse10/2019/en#/II; 2019 [accessed 7 July 2022].

[14] WHO collaborating centre for drug statistics methodology, ATC/DDD Index 2022, https://www.whocc.no/atc_ddd_index/; 2021 [accessed 7 July 2022].

[15] The German centre for cancer registry data (ZfKD), https://www.krebsdaten.de/Krebs/SiteGlobals/Forms/Datenbankabfrage/EN/datenbankabfrage_stufe1_form.html [accessed 13 July 2022].

[16] Yang S, Song Z, Cheng G. Genomic alterations and survival in young patients aged under 40 years with completely resected non-small cell lung cancer. Ann Transl Med. 2019;7:140. 10.21037/atm.2019.03.39. 31157261 10.21037/atm.2019.03.39 PMC6511547

[17] Maletzki C, Hühns M, Bauer I, Prall F, Junghanss C, Henze L. Suspected hereditary cancer syndromes in young patients: heterogeneous clinical and genetic presentation of colorectal cancers. Oncologist. 2019;24:877–82. 10.1634/theoncologist.2018-0614. 30683709 10.1634/theoncologist.2018-0614 PMC6656443

[18] Pearlman R, Frankel WL, Swanson B, et al. Prevalence and spectrum of germline cancer susceptibility gene mutations among patients with early-onset colorectal cancer. JAMA Oncol. 2017;3:464–71. 10.1001/jamaoncol.2016.5194. 27978560 10.1001/jamaoncol.2016.5194 PMC5564179

[19] Wang Y, Chen J, Ding W, et al. Clinical features and gene mutations of lung cancer patients 30 years of age or younger. PLoS ONE. 2015;10:e0136659. 10.1371/journal.pone.0136659. 26332764 10.1371/journal.pone.0136659 PMC4557988

[20] Internisten im Netz, UICC-Stadien, https://www.internisten-im-netz.de/glossar/begriff/uicc-stadien.html [accessed 7 July 2022], [in German].

[21] Onko Internetportal, Klassifikation von Tumoren (TNM-System & Grading), https://www.krebsgesellschaft.de/onko-internetportal/basis-informationen-krebs/basis-informationen-krebs-allgemeine-informationen/klassifikation-von-tumoren-tnm-.html; 2015 [accessed 7 July 2022], [in German].

[22] Oken M, Creech R, Tormey D, et al. Toxicity and response criteria of the Eastern cooperative oncology group. Am J Clin Oncol. 1982;5(6):649–55. 7165009

[23] Bayerisches Landesamt für Gesundheit und Lebensmittelsicherheit (LGL), Bavarian Cancer Registry, https://www.lgl.bayern.de/gesundheit/krebsregister/index_e.htm; 2022 [accessed 28 July 2022].

[24] Ke G, Meng Q, Finley T, Wang T, Chen W, Ma W, Ye Q, Liu TY. LightGBM: a highly efficient gradient boosting decision tree. Adv Neural Inf Process Syst. 2017;30:3149–57.

[25] Borisov V, Leemann T, Seßler K, Haug J, Pawelczyk M, Kasneci G. Deep neural networks and tabular data: A survey. 2021. 10.48550/arXiv.2110.01889. Preprint arXiv:2110.01889 10.1109/TNNLS.2022.3229161 37015381

[26] Mienye ID, Sun Y. Performance analysis of cost-sensitive learning methods with application to imbalanced medical data. Inf Med Unlocked. 2021;25:100690.

[27] Pedregosa F, Varoquaux G, Gramfort A, et al. Scikit-learn: machine learning in python. JMLR. 2011;12:2825–30.

[28] LightGBM, Parameters tuning, https://lightgbm.readthedocs.io/en/latest/Parameters-Tuning.html [accessed 20 July 2022].

[29] Zou Q, Xie S, Lin Z, Wu M, Ju Y. Finding the best classification threshold in imbalanced classification. Big Data Res. 2016;5:2–8. 10.1016/j.bdr.2015.12.001.

[30] Powers DMW. Evaluation: from precision, recall and f-measure to roc, informedness, markedness & correlation. J Mach Learn Technol. 2011;2:37–63.

[31] Zhao Y, Wong ZS, Tsui KL. A framework of rebalancing imbalanced healthcare data for rare events’ classification: a case of look-alike sound-alike mix-up incident detection. J Healthc Eng. 2018;2018:6275435. 29951182 10.1155/2018/6275435 PMC5987310

[32] Hicks SA, Strümke I, Thambawita V, et al. On evaluation metrics for medical applications of artificial intelligence. Sci Rep. 2022;12:5979. 35395867 10.1038/s41598-022-09954-8 PMC8993826

[33] Lu H, Xu Y, Ye M, et al. Learning misclassification costs for imbalanced classification on gene expression data. BMC Bioinf. 2019;20:681. 10.1186/s12859-019-3255-x. 10.1186/s12859-019-3255-x PMC6929277 31874599

[34] Shegai PV, Shatalov PA, Zabolotneva AA, Falaleeva NA, Ivanov SA, Kaprin AD. Challenges faced by clinicians in the personalized treatment planning: a literature review and the first results of the Russian national cancer program. Crit Care Res Pract. 2021;2021:6649771. 34603796 10.1155/2021/6649771 PMC8483928

[35] Schwarze K, Buchanan J, Fermont JM, et al. The complete costs of genome sequencing: a microcosting study in cancer and rare diseases from a single center in the United Kingdom. Genet Med. 2020;22:85–94. 10.1038/s41436-019-0618-7. 31358947 10.1038/s41436-019-0618-7 PMC6944636

[36] Kumar S, Bennett A, Campbell PA, et al. Costs of next-generation sequencing assays in non-small cell lung cancer: a micro-costing study. Curr Oncol. 2022;29:5238–46. 10.3390/curroncol29080416. 35892985 10.3390/curroncol29080416 PMC9330154

[37] McCombie WR, McPherson JD. Future promises and concerns of ubiquitous next-generation sequencing. Cold Spring Harb Perspect Med. 2019;9:a025783. 10.1101/cshperspect.a025783. 30478095 10.1101/cshperspect.a025783 PMC6719590

[38] Nonnemacher M, Nasseh D, Stausberg J. Datenqualität in der medizinischen Forschung: Leitlinie zum adaptiven Management von Datenqualität in Kohortenstudien und Registern. Berlin: Medizinisch Wissenschaftliche Verlagsgesellschaft; 2014.

[39] Borner M, Schweizer D, Fey T, Nasseh D, Dengler R. A Source data verification-based data quality analysis within the network of a german comprehensive cancer center. Transdisciplinary perspectives on public health in Europe. Springer Gabler 2022; 189–200. 10.1007/978-3-658-33740-7_11

[40] Hemkens LG, Contopoulos-Ioannidis DG, Ioannidis JPA. Routinely collected data and comparative effectiveness evidence: promises and limitations. CMAJ. 2016;188:E158–64. 10.1503/cmaj.150653. 26883316 10.1503/cmaj.150653 PMC4868623

[41] Kim GYE, Noshad M, Stehr H, et al. Machine learning predictability of clinical next generation sequencing for hematologic malignancies to guide high-value precision medicine. AMIA Annu Symp Proc. 2022;2021:641–50. 35308914 PMC8861666

[42] Deutsches Netzwerk für Personalisierte Medizin (DNPM), https://dnpm.de/[accessed 23 September 2022], [in German].

[43] German cancer consortium (DKTK), current projects: molecular tumor board alliance (MTBA), https://dktk.dkfz.de/en/research/joint-funding-projects/current-projects [accessed 23 September 2022].

[44] The Bavarian cancer research center (BZKF), https://bzkf.de/?lang=en [accessed 23 September 2022].

[45] Medical informatics initiative Germany (MII), https://www.medizininformatik-initiative.de/en/start [accessed 23 September 2022].

